# An incidentally identified 15 cm cavernous hemangioma of the small intestine: Case report and literature review

**DOI:** 10.1016/j.ijscr.2021.106144

**Published:** 2021-06-29

**Authors:** Takeshi Kano, Shota Fukai, Risa Okamoto, Yasuaki Motomura, Alan Kawarai Lefor, Ken Mizokami

**Affiliations:** a Department of Surgery, Tokyo Bay Urayasu-Ichikawa Medical Center, 3-4-32 Todaijima, Urayasu, Chiba 279-0001, Japan; b Department of Gastroenterology, Tokyo Bay Urayasu-Ichikawa Medical Center, 3-4-32 Todaijima, Urayasu, Chiba 279-0001, Japan; c Department of Surgery, Jichi Medical University, 3311-1 Yakushiji, Shimotsuke-shi, Tochigi, 329-0498, Japan

**Keywords:** Hemangioma of the small intestine, Laparoscopic surgery, Minimally invasive surgery, Case report

## Abstract

**Introduction:**

Hemangiomas of the small intestine are rare, usually present with symptoms such as anemia, gastrointestinal bleeding or abdominal pain and are resected. We report resection of an incidentally identified cavernous hemangioma of the small intestine that did not present symptoms referable to the hemangioma. Although it was a large lesion, it was resected using laparoscopy and a mini-laparotomy.

**Presentation of case:**

A 29-year-old otherwise healthy man was referred for evaluation of ileal wall thickening found on a contrast-enhanced computed tomography scan obtained for the workup of chronic diarrhea. Double balloon enteroscopy (DBE) showed a cavernous hemangioma of the small intestine. The lesion was 15 cm and resected using laparoscopy and a mini-laparotomy to prevent future bleeding. The histopathological diagnosis was a cavernous hemangioma of the ileum.

**Discussion:**

Though there have been no reports of the asymptomatic patients of the disease, the recent spread of double balloon enteroscopy and capsule endoscopy will allow us to diagnose more asymptomatic hemangiomas like this patient. Also, this large lesion was able to be resected through a small incision due to its compressible nature.

**Conclusion:**

Future studies in asymptomatic patients of the disease may help to determine the optimal management for these patients. Even large hemangiomas are compressible, facilitating minimally invasive resection.

## Highlights

• Hemangiomas of the small intestine are rare disease.
• They have always been diagnosed with symptoms such as anemia and bleeding so far.
• An asymptomatic hemangioma of the small intestine was incidentally identified.
• It was big, but laparoscopic resection was possible due to its compressible nature.
• To determine the optimal treatment for asymptomatic lesions is a task in the future.

## 1 Introduction

Hemangiomas of the small intestine are rare and usually identified when the patient presents with symptoms such as anemia, gastrointestinal bleeding or abdominal pain [1]. Resection is the treatment of choice for symptomatic lesions [1]. According to our literature review, all patients previously reported were symptomatic.

A 15 cm hemangioma of the small intestine was found incidentally without typical presentations of the disease. Though it was a large lesion, resection using a minimally invasive approach with laparoscopy and mini-laparotomy was possible. This work is reported in line with the SCARE 2020 criteria [2].

## 2 Presentation of case

**Fig. 1:**
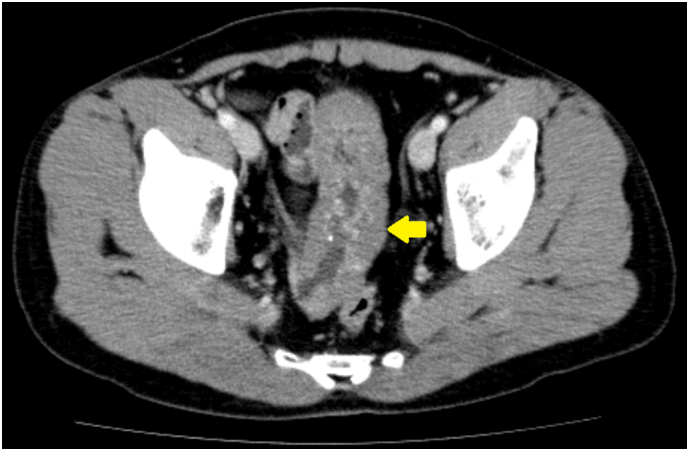
Preoperative computed tomography (CT) scan. Contrast-enhanced abdominal CT scan in a 29-year-old man who presented with chronic diarrhea, showed a 15 cm irregular thickening in the ileum, with focal calcifications.

**Fig. 2:**
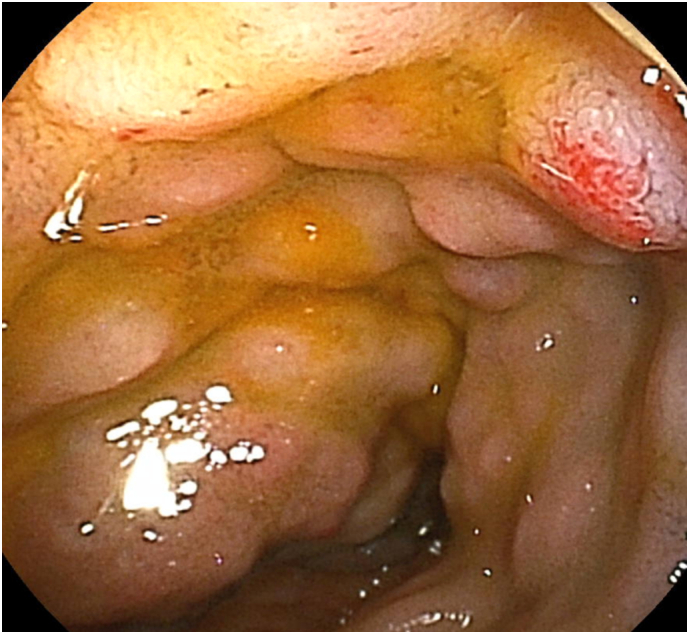
Endoscopic appearance of the lesion. Double balloon enteroscopy revealed multiple reddish purple submucosal masses in the distal ileum. (For interpretation of the references to colour in this figure legend, the reader is referred to the web version of this article.)

A 29-year-old healthy man was referred because a contrast-enhanced computed tomography scan obtained to evaluate a two-year history of diarrhea showed wall thickening in the ileum (Fig. 1). The patient had no history of gastrointestinal bleeding. Physical examination showed a blood pressure of 139/106 mmHg, heart rate of 82 beats/min, and normal temperature. Abdominal examination showed no tenderness. Laboratory tests showed no anemia (hemoglobin 16.3 g/dl). For further investigation, DBE was performed, which revealed multiple red-purple submucosal masses in the distal ileum (Fig. 2). Based on endoscopic findings, this was thought to be a cavernous hemangioma. The relationship of diarrhea to the hemangioma was not clear but considering the patient's young age, surgical resection was recommended to prevent bleeding in the future.

**Fig. 3:**
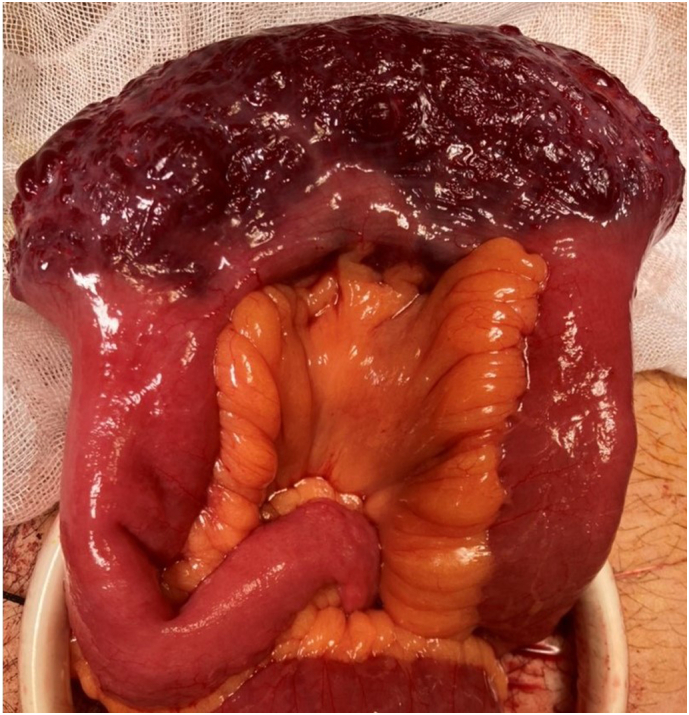
Gross appearance of the lesion. A 15 cm × 6 cm red-purple lesion along the serosal surface in the ileum. (For interpretation of the references to colour in this figure legend, the reader is referred to the web version of this article.)

**Fig. 4:**
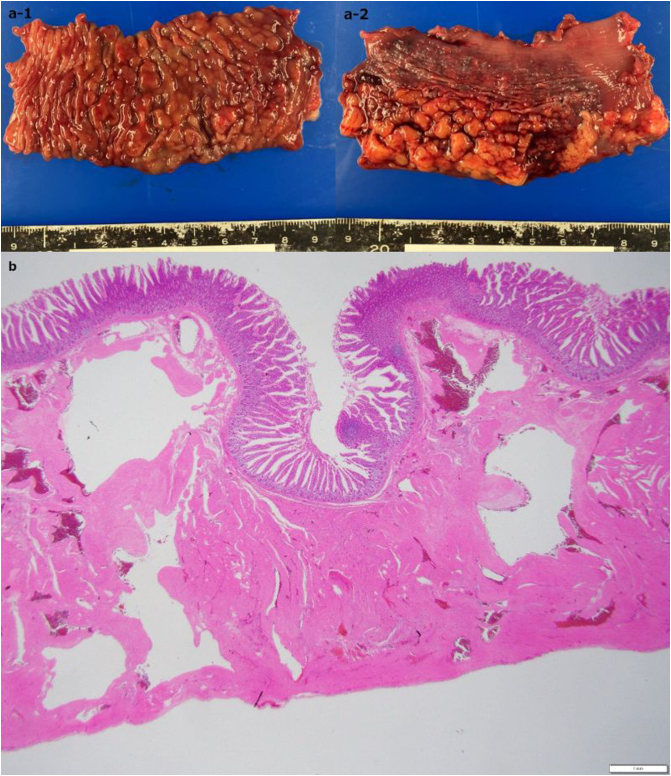
Macroscopic appearance and histological examination of the resected specimen. a. Multiple submucosal masses(a-1: mucosal surface, a-2: serosal surface). The lesion shrunk immediately after resection. b. Sinus-like spaces in the submucosa and muscularis propria (hematoxylin and eosin staining, × 40). Some of these spaces were filled with blood.

Diagnostic laparoscopy was performed first, which revealed a 15 cm × 6 cm red-purple lesion along the serosal surface of the ileum. The lesion was easily extracted manually through a 5 cm incision and a functional end-to-end anastomosis performed after resection (Fig. 3). Immediately after resection, the lesion shrunk to 10 cm × 3 cm suggesting its vascular nature (Fig. 4a). The histopathological diagnosis was a cavernous hemangioma of the ileum (Fig. 4b). The postoperative course was uneventful, and he was discharged on postoperative day five. Two weeks postoperatively, the chronic diarrhea was unchanged, suggesting that the hemangioma was not the cause of his symptom.

## 3 Discussion

There were two notable things regarding this patient. First, the patient had no symptoms referable to the hemangioma. Second, this large lesion was resected through a small incision.

In order to review previous reports, PubMed was searched for reports of hemangiomas of the small intestine published after 2000 using the following search terms: “hemangioma”, “small intestine” and “small bowel”. A manual search was then performed based on the references from eligible articles. The language was limited to English. A total of 45 cases were retrieved and reviewed. Most of the 45 patients presented with anemia, gastrointestinal bleeding or abdominal pain. No lesions were found incidentally, and no patient presented with diarrhea.

The relationship between diarrhea and the hemangioma in the present patient cannot be known with certainty but given that the diarrhea persisted after resection and that no previously reported patients had diarrhea, the diarrhea had another etiology and the hemangioma was not causing symptoms. Among patients previously reported, none of them were asymptomatic, and we felt that operative resection was indicated to prevent future bleeding. Endoscopic therapy is one of the treatment options and among 45 cases we reviewed, it was performed for 3 lesions [3], [4]. The sizes of these lesions were 0.8 cm, 1.4 cm and 2 cm, respectively and endoscopic therapy tends to be applied for small lesions. So we chose not endoscopic treatment but operative resection.

Diagnosis of hemangiomas of the small intestine was difficult in the past because there was no way to view the small intestinal mucosa non-invasively. DBE and capsule endoscopy are now widely available and the lumen of the small intestine can be easily imaged. This suggests that there may be more reports of asymptomatic patients with small bowel lesions such as hemangiomas in the future, and the optimal management of these patients will be established.

This 15 cm lesion was resected through a small incision in this patient. The diameter of previously reported lesions varied from 0.8 cm to 50 cm with an average size of 7.0 ± 8.8 cm. The lesion in this patient is larger than most but was easily extracted manually through a 5 cm incision. This is likely due to the compressible nature of a hemangioma since they are not true solid tumors but just venous malformations. This large lesion compressed easily at the time of extraction like sponges. In the literature review, there were 3 lesions greater than 15 cm [5], [6], [7]. We do not know the size of the incision used in these reports, but based on our experience, lesions larger than 15 cm can be resected in a minimally invasive way.

## 4 Conclusion

An incidentally identified 15 cm hemangioma of the small intestine was resected to prevent future bleeding. Previous reports have only included symptomatic patients, but the advent of imaging of the small intestinal mucosa may lead to the identification of more such lesions. Future studies in asymptomatic patients may help to determine the optimal management for these patients. Even large hemangiomas of the small intestine are compressible, facilitating minimally invasive resection.

## Funding

Authors had no sources of funding.

## Ethical approval and consent to participate

Not applicable.

## Consent

Informed consent for the publication of this work was given by the patient. Written informed consent was obtained from the patient for publication of this case report and accompanying images. A copy of the written consent is available for review by the Editor-in-Chief of this journal on request.

## Registration of research studies

Not applicable.

## Guarantor

Takeshi Kano MD.

## Provenance and peer review

Not commissioned, externally peer-reviewed.

## CRediT authorship contribution statement

TK and KM gathered the patient's data and wrote the manuscript. KM was responsible for the in-patient optimization. SF, RO, YM, AKL and KM reviewed the manuscript. All authors approved the final manuscript.

## Declaration of competing interest

There are no conflicts of interest to be declared.
